# Supplementary figures and images for: Efficacy and immunogenicity of rKVAC85B in a BCG prime-boost regimen against H37Rv and HN878 Mycobacterium tuberculosis strains
Source: PLoS One. 2025 May 14;20(5):e0322147. doi: 10.1371/journal.pone.0322147 (PMC12077692; doi:10.1371/journal.pone.0322147)

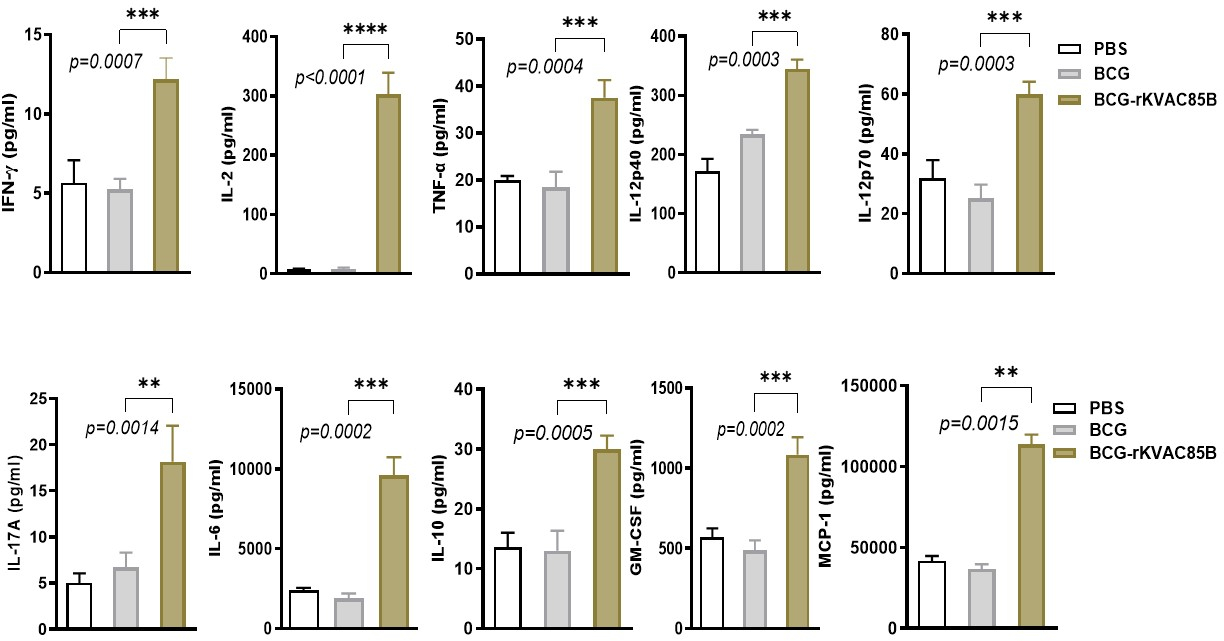

Supplement: S1 Fig — Cytokine responses in the pulmonary compartment were quantified following a prime-boost vaccination regimen using bead-based ELISA. Lung cells were harvested and stimulated with PPD (WHO international standard purified protein derivative of Mycobacterium tuberculosis, National Institute of Biological Standards and Control (NIBSC), 100 ng/well) for 36 h to assess post-vaccination cytokine levels. This comparative analysis delineated cytokine induction across three groups: PBS control, BCG priming alone, and BCG priming followed by rKVAC85B booster. The cytokines measured include IFN-γ, IL-2, TNF-α, IL-12p40, IL-12p70, IL-17A, IL-6, IL-10, GM-CSF, and MCP-1. Results are expressed as mean ± standard deviation (SD), and levels of statistical significance are marked with asterisks: **p < 0.01, ***p < 0.001, indicating a significant increase in the cytokine levels in the BCG and rKVAC85B co-immunized groups. (TIF) [file pone.0322147.s001.tif]

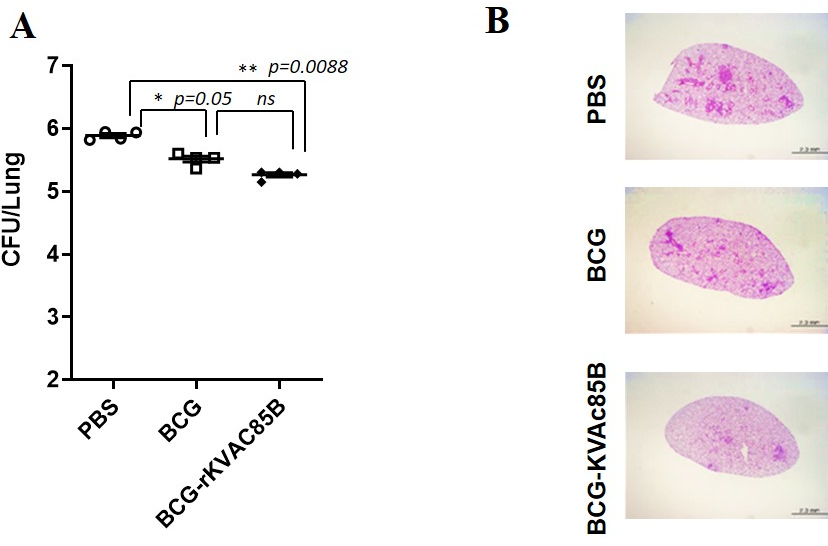

Supplement: S2 Fig — Bacterial-load quantification in the lungs of mice, following an immunization schedule of BCG priming and subsequent rKVAC85B boosts, was performed to determine the immunoprotective effects against two distinct strains of M. tuberculosis. (A) H37Rv strain challenge: In an experimental setup similar to that in Fig 7 (A) HN878 strain challenge: CFUs in the lung tissues were counted 8 eight weeks to determine the strain-specific protective efficacy of the vaccine regimen. (B) display histological sections of the lung tissues stained with hematoxylin and eosin (H&E) post-infection with H37Rv strain. The scale bar represents 2 μm. Statistical significance of differences in CFUs between the groups was determined using the one-way ANOVA, with p-values indicating the level of significance, where *p ≤ 0.05, **p ≤ 0.01, and ***p ≤ 0.001. “ns” denotes not significant. (TIF) [file pone.0322147.s002.tif]

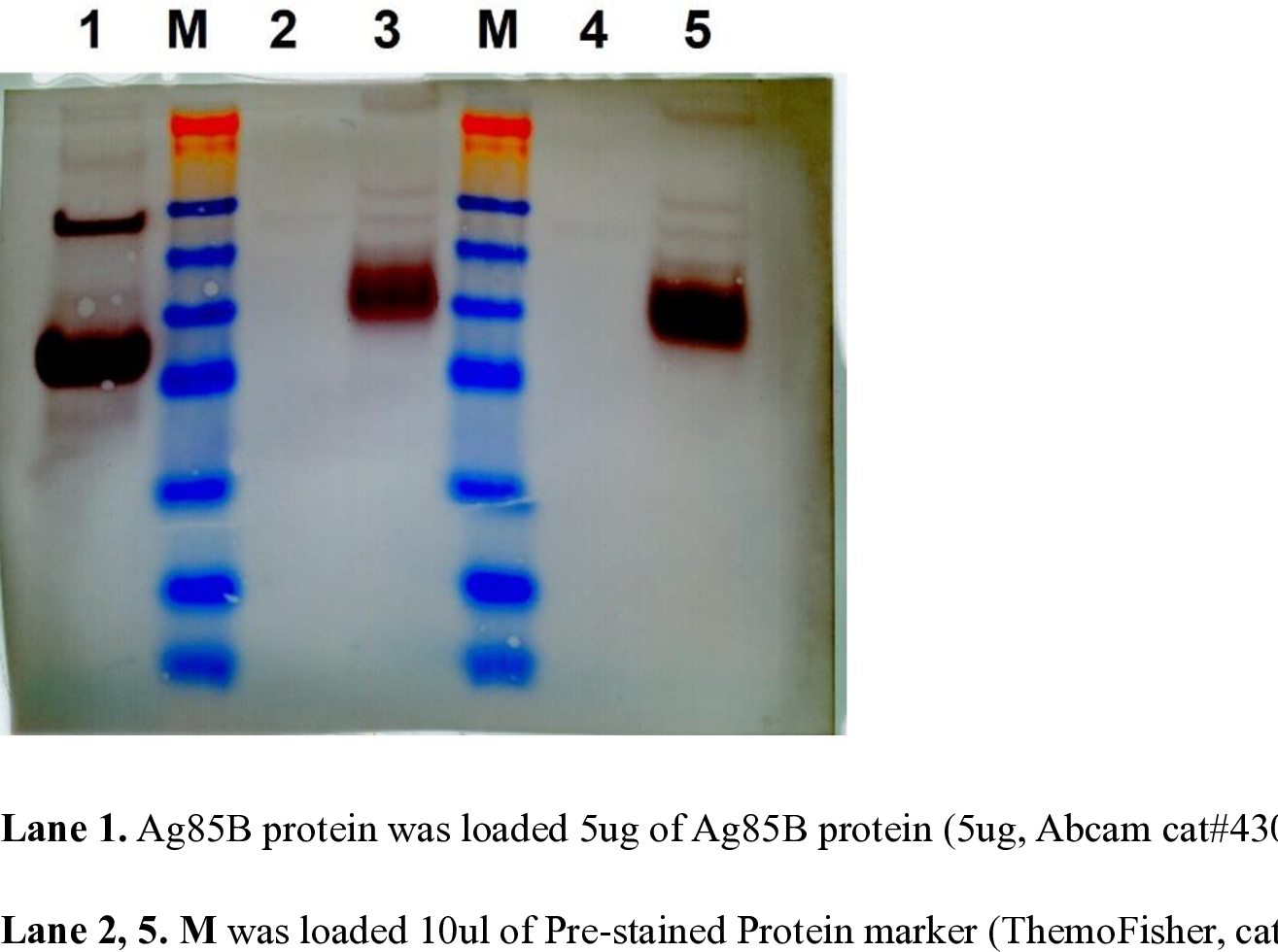

Supplement: S1 Raw Data Gel Image — (TIF) [file pone.0322147.s003.tif]
