# Supplementary material for: Efficacy and immunogenicity of rKVAC85B in a BCG prime-boost regimen against H37Rv and HN878 Mycobacterium tuberculosis strains
Source: PLoS One. 2025 May 14;20(5):e0322147. doi: 10.1371/journal.pone.0322147 (PMC12077692; doi:10.1371/journal.pone.0322147)
Supplement: Attachment — Submitted filename: Reviewers_comment_PONE-D-24-18487.pdf [file pone.0322147.s005.pdf]

Reviewer's Comments on Manuscript PONE-D-24-18487

Title: Efficacy and Immunogenicity of rKVAC85B in a BCG Prime-Boost Regimen Against H37Rv and HN878 Mycobacterium Tuberculosis Strains

General Assessment:
The manuscript presents a study on the efficacy and immunogenicity of a recombinant vaccinia virus expressing Ag85B of *M. tuberculosis* (rKVAC85B) employing two prime-boost regimens against two strains of *M. tuberculosis:* standard laboratory H37Rv and the hypervirulent HN878 strains. The research is timely and addresses a critical need for improved tuberculosis vaccines, especially considering the variable efficacy of BCG in adults.

Strengths:
The study introduces a novel vaccine candidate using KVAC103, an attenuated vaccinia virus as vaccine vector to express Ag85B, which is of significant interest in the field of TB vaccine research. The focus on both H37Rv and hypervirulent HN878 strains enhances the study's relevance. The study adheres to ethical standards, with appropriate approvals and descriptions of animal welfare considerations.

Overall Recommendation:
This manuscript presents advancements in TB vaccine research and may be considered for publication after addressing and revising the following points thoroughly.

Areas for Improvement:

1. **Introduction:**
   While the existing introduction provides a wide background but could benefit from more detailed but specific information on the limitations of BCG and other current TB vaccines in clinical trial, and the specific advantages of using vaccinia virus vectors and the specific antigen (Ag85B) and its specific role in *M. tuberculosis*.

2. **Materials and Methods:**
   The M&M section requires further comprehensive details. For instance:
   (i) the description of the generation of rKVAC85B could include more details on the verification steps (e.g., PCR conditions, primer sequences, and agarose gel images) as supporting data, such that it should be easy to follow the protocols by other researcher or reader.
   (ii) the authors should provide clear pictorial (cartoon) outlines of the immunogenicity and protective efficacy study protocols as part of the respective result figures, for greater clarity to the reader.
   (iii) the methodology for the bead-based cytokine analysis could be expanded to include the rationale for selecting these cytokines.
   (iv) while MGIA is an interesting and powerful *ex vivo* assay to measure the efficacy of cellular immunity in controlling bacterial growth, inclusion of BCG as the target bacteria reduces its significance in this study that targets to evaluate the efficacy of new vaccine against two virulent strains of *M. tuberculosis***.** This requires appropriate rationale or redoing it with virulent *M. tuberculosis*.

3. **Results:**

The description of results and the respective figures need appropriate revision. For instance:

(i) the immunogenicity experiment seems to be conducted for all five groups together (Unvaccinated, BCG, rKVAC85B, rKVAC85B prime boost, and BCG- KVAC85B prime boost) as described in the M&M section (Line 150). However, results were depicted separately which makes it difficult to compare among all the groups. Animal numbers in each group for immunogenicity study was not mentioned, while for efficacy study it is mentioned as (n=5). Figure 2 C and D, and Figure 4 A and B bar diagrams should be provided with the error bars and appropriate statistics to show significance of the observed differences. The conc. of Ag85B protein that was used as the coating/stimulating agent not mentioned. Details of Ag85B epitope/peptides used for ELISPOT assay should be provided. Figure 5, IL-10 levels were depicted, typically this is a Th2 cytokine and known to enhance pathogen favoured response in TB, however, its heightened induction in the case of BCG prime rKVAC85B was not explained well. Information on number of samples, technical replicates should be provided.

(ii) as pointed out above, use of BCG as the target pathogen in the MGIA requires justification.

(iii) typically, in the mouse model of TB vaccine efficacy studies, both lungs and spleen CFU counts are provided. In this study, only lung CFU was shown. Speen CFU data highlights how the vaccination would prevent dissemination of infecting pathogen to distant lymphoid organ. It would be better to show both lung and spleen CFU data. Further, in the absence of details of lungs homogenisation and plating protocol (which/how many lobes for what purpose, weight of lungs) it is difficult to appreciate the CFU data presentation as Log10 CFU/ml as weight/size of lungs varies between vaccinated and unvaccinated animals. Figure 7 needs major revision, it is appropriate to depict CFU data as per lungs or per gram of tissue, CFU data for all the groups in the study is warranted, labelling of the unvaccinated and infected group as normal is technically incorrect, should be revised.

4. **Discussion:**

The discussion could be strengthened by more critically comparing the results with existing studies on TB vaccines, particularly those using similar viral vectors, and explaining why the vaccine under study could be superior. Discuss the conundrum of heightened L-10 response in the study. Also, highlight future research directions.

Minor Comments:

(i) Ensure consistency in the use of abbreviations throughout the manuscript.
(ii) A few typographical and grammatical errors need correction for clarity and readability.
(iii) Ensure all references are up-to-date and formatted according to the journal's guidelines.
