# Supplementary material for: Efficacy and immunogenicity of rKVAC85B in a BCG prime-boost regimen against H37Rv and HN878 Mycobacterium tuberculosis strains
Source: PLoS One. 2025 May 14;20(5):e0322147. doi: 10.1371/journal.pone.0322147 (PMC12077692; doi:10.1371/journal.pone.0322147)
Supplement: Attachment — Submitted filename: Reviewers_comment_final_20241118.docx [file pone.0322147.s007.docx]

**Response to Reviewer`s Comments**

**Reviewer-1`s Comments to Author**

The authors describe a novel vaccinal construct for vaccination against Tuberculosis. It is a novel attenuated vaccinia virus (KVAC103) expressing Ag85B (rKVAC85B). They analyzed the immunogenicity and the efficacy of the vaccine, also in a prime-boost inoculation strategies with BCG in a murine model of TB. the work is quite interesting even if not very original, unfortunately it has serious experimental limitations.

Major points:
1. In all the figures 2, 3, 4 e 5 only data "specific" for Ag85B are reported. The baseline responses (e.g. only culture medium) are missing. These controls are required to evaluate the baseline activation of cells and the increase/decrease due to Ag85B restimulation.

Reply: Thank you for your valuable feedback. In our study, we have already included results from the PBS control group, where both IgG and Elispot assays showed negligible or no response. This indicates a very low baseline activation level of cells under our experimental conditions when not stimulated by Ag85B, thus confirming that additional baseline control data may not be necessary to assess the effects of Ag85B accurately. We believe our existing data robustly supports the conclusions drawn and are confident in its objectivity and validity. Should you have further comments or require additional information, please do not hesitate to contact us. Thank you very much for your attention and guidance.

2- In Figure 2, the data related to the vaccination with BCG+rKVAC85B alone are missing. Is the prime with BCG enhances, reduces or leaves in altered the Ag85B responses induced by rKVAC85B alone?

Reply: Thank you once again for your meticulous comments and suggestions. Analyzing the combined administration of BCG with rKVAC85B is indeed an intriguing research direction, and exploring such priming vaccination strategies could be highly valuable for future studies.

However, the current study was specifically designed to focus on the potential of rKVAC85B as an alternative or booster vaccine to BCG. Accordingly, our research concentrated on scenarios where rKVAC85B was administered either alone or following BCG vaccination. Investigating the concurrent use of BCG and rKVAC85B falls beyond the scope of this study and would be more appropriately addressed in subsequent research.

Your suggestion will be an important reference for setting future research directions, and we will consider this point when planning further studies. Thank you once again for your valuable feedback and suggestions.

3- In all the figures 3, 4 e 5 the data related to the control vaccination with rKVAC85B alone are missing. An experiment in which all the various vaccine combinations are present at the same time is necessary to be able to make adequate comparisons. The data are required to explain the data showed in Figure 6 and Figure 7 (MGIA and in vivo MTB infection). Is the prime with BCG enhances, reduces or leaves in altered the Ag85B responses induced by rKVAC85B alone?

Reply: Thank you once again for your meticulous observations and suggestions. In our study, Figures 3 through 7 have presented results from a subsequent vaccination with rKVAC85B following a BCG prime. Notably, the results displayed in Figures 3B and 3C show a significant increase in both IgG and IFN-γ responses when compared to the rKVAC85B-alone group (Figures 2B and 2C). These findings served as a foundation for us to further investigate the boosting effects of BCG. Consequently, to more precisely analyze the effects of a BCG primed rKVAC85B boost, we conducted MGIA experiments (Figure 6) and *in vivo* MTB infection tests (Figure 7). The enhanced immune responses observed following the sequential administration of BCG and rKVAC85B provide critical insights into current vaccine strategies that utilize BCG as a primary vaccination.

4- Spleen cells of BCG-vaccinated mice are not good responder to Ag85B protein; ex vivo Ag85B restimulation can even inhibit the basal cytokine production by cells of BCG-vaccinated mice. Therefore, knowing not only the basal level of cytokine production but also that induced by stimulation with other MTB antigens, such as PPD, is essential to understand how the vaccine rKVAC85B modulates the entire BCG-mediated immune response.

Reply: Thank you once again for your invaluable feedback. In response to your comments, we have included the bead-based cytokine assay results using PPD antigen in Supporting Information Fig S1. These results demonstrate a similar increase in cytokine secretion as observed in Figure 5, where Ag85B peptide was employed. Therefore, it is anticipated that the ten cytokines examined in this study may be similarly secreted in response to stimulation by other MTB antigens beyond Ag85B.

The additional results have been incorporated into the manuscript at lines 427-431.

**S1 Fig. Measurement of pulmonary cytokine response by PPD following BCG prime-rKVAC85B boost.**

5- Evaluation of Ag85B-specific immune responses after 1 week from the last immunization is not idoneous to reveal memory immunity, which is a priority to demonstrate the vaccine efficacy. Longer times need to be studied.6- In MGIA experiments (Figure 6), as well as in in vivo experiments (Figure 7) the data related to vaccination with rKVAC85B alone are missing. Is vaccination with rKVAC85B alone protective?

Reply: Thank you once again for your meticulous observations and suggestions. In response to your comments regarding the timing of immune response evaluations, we acknowledge the challenges inherent in analyzing long-term memory immunity. While our experimental timeline of assessing immune responses 7-10 days post-final immunization is aligned with common schedules used in other studies (PMID: 25070842, 26599077, 22921259, and 36845108) to analyze the boosting effects, we recognize, as you have rightly pointed out, that this approach may be insufficient for evaluating long-term memory immunity comprehensively. We appreciate your insights into the necessity for assessing long-term memory immunity and agree that a longer timeframe is necessary to accurately evaluate the memory response. Thank you once again for your thorough review and for aiding in the enhancement of our study's quality.

6-In MGIA experiments (Figure 6), as well as in in vivo experiments (Figure 7) the data related to vaccination with rKVAC85B alone are missing. Is vaccination with rKVAC85B alone protective?

Reply: Thank you once again for your meticulous observations and insightful comments. In our study, the IgG and IFN-γ response data presented in Figures 3B and 3C served as a crucial basis for analyzing the effects of rKVAC85B boosting following BCG priming. This analysis directed us to undertake MGIA experiments (Figure 6) and in vivo MTB infection tests (Figure 7). Unfortunately, the specific focus of these experiments led to the inadvertent omission of data regarding the solitary administration of rKVAC85B, for which we kindly request your understanding. We fully acknowledge the importance of assessing rKVAC85B as a standalone vaccine, as you have rightly highlighted. Such an evaluation is indeed critical for a comprehensive understanding of its efficacy. However, the primary aim of our current study is to investigate the synergistic effects that arise from combining BCG with rKVAC85B. Our research is driven by the hypothesis that this combination might significantly enhance immune responses, potentially offering superior protection compared to the vaccines administered individually.

7- In MGIA experiments (Figure 6), the “potential” presence of BCG in spleen cells of BCG- or BCG+rKVAC85B-vaccinated mice, prior to in vitro BCG infection, needs to be evaluated. Do the spleens of mice vaccinated with BCG or BCG+rKVAC85B harbor live BCG at the time of organ explantation?

Reply: Thank you for your insightful query regarding the MGIA experiments detailed in Figure 6 of our manuscript. Your question concerning the potential presence of live BCG in the spleen cells of mice vaccinated with BCG or BCG+rKVAC85B at the time of organ explantation is crucial for accurate interpretation of the experimental outcomes. We have previously assessed the presence of residual BCG in the spleens of vaccinated mice by culturing spleen cells from the BCG-vaccinated group alone (without co-culturing with BCG strains) to check for time to detection (TTD) of any viable BCG. Our findings indicated that TTD was not achievable, suggesting the absence of viable BCG in the spleen cells at the time of organ collection. This provides a strong indication that the spleens of these mice do not harbor live BCG at the time of explantation.

8- In Figure 7, why the CFU are higher in the lungs of mice infected with H37Rv instead of those infected with the hypervirulent HN878 strain? Furthermore, histology data should be graphed and an appropriate statistical analysis performed to demonstrate that the inflammation and area occupied by granulomas are higher in BCG- than in BCG+rKVAC85B-vaccinated mice.

Reply: Thank you for your insightful query about Figure 7. The higher CFU counts in the lungs of mice infected with H37Rv compared to the hypervirulent HN878 strain at 8 weeks post-infection align with previous studies (PMID: 31832640). Despite HN878's hypervirulence, it elicits a strong Th1 immune response that effectively reduces the bacterial load but causes significant tissue damage. In contrast, H37Rv induces a milder immune response, allowing higher bacterial persistence and resulting in higher CFU counts. These findings emphasize the complex interaction between bacterial virulence and host immune responses. Therefore, we have focused our revised Figure 7 on the outcomes in HN878-infected mice and included H37Rv data in Supporting Figure 2, highlighting the different impacts on vaccine efficacy.

**Fig 7. Comparative efficacy of BCG prime-rKVAC85B boost immunization against *M. tuberculosis* HN878.**

**S2 Fig. Immunoprotective Efficacy of BCG prime-rKVAC85B boosted against H37Rv.**

9-In the Discussion, lines 398-417 and 420-444 are appropriate for the Introduction section.

Reply: Thank you for your valuable feedback. In response to your insights, we have added relevant content to Lines 69-88 and Lines 107-128 of our manuscript.

10-in the Discussion, it is reported that “the increase in the IFN-γ levels following rKVAC85B vaccination highlights the vaccine's potential to elicit a strong immune defense against intracellular infections”, lines 449-450. Although the protective role of IFN-γ in TB is undoubted, many papers indicate that these cytokines needs to be balanced and its enhancement does not always correlate with better protection against Mtb infection. It is also true for the IFN-γ generated in response to Ag85B protein. This point should be discussed with greater attention also considering that there is no data in the work which supports the relationship between the increase in IFN-γ and protection.

Reply: Thank you for your valuable feedback. In response to your insights, we have added relevant content to Lines 79-88 and Lines 107-128 of our manuscript.

Minor points
1- Add refs in the introduction section line 82

Reply: In response to your guidance, we have incorporated the additional references at Line 134 of the manuscript.

**Reviewer-2`s Comments to Author**

General Assessment:
The manuscript presents a study on the efficacy and immunogenicity of a recombinant vaccinia virus expressing Ag85B of M. tuberculosis (rKVAC85B) employing two prime-boost regimens against two strains of M. tuberculosis: standard laboratory H37Rv and the hypervirulent HN878 strains. The research is timely and addresses a critical need for improved tuberculosis vaccines, especially considering the variable efficacy of BCG in adults.

Strengths:
The study introduces a novel vaccine candidate using KVAC103, an attenuated vaccinia virus as vaccine vector to express Ag85B, which is of significant interest in the field of TB vaccine research. The focus on both H37Rv and hypervirulent HN878 strains enhances the study's relevance. The study adheres to ethical standards, with appropriate approvals and descriptions of animal welfare considerations.

Areas for Improvement:
1. Introduction:
While the existing introduction provides a wide background but could benefit from more detailed but specific information on the limitations of BCG and other current TB vaccines in clinical trial, and the specific advantages of using vaccinia virus vectors and the specific antigen (Ag85B) and its specific role in M. tuberculosis.
Reply: Thank you for your valuable comments. In response to your suggestion, we have further refined the introduction section of our manuscript by incorporating more detailed and specific information about the limitations of BCG and other current TB vaccines in clinical trials, and the distinct advantages of using vaccinia virus vectors and the specific antigen Ag85B. (line 134-148)

2. Materials and Methods:
The M&M section requires further comprehensive details. For instance:
(i) the description of the generation of rKVAC85B could include more details on the verification steps (e.g., PCR conditions, primer sequences, and agarose gel images) as supporting data, such that it should be easy to follow the protocols by other researcher or reader.

Reply: In response to your suggestions regarding the detailed description of the rKVAC85B generation process, we have included additional information in the Methods section of our manuscript (line 192-198).

Specifically, the primer information and PCR conditions are detailed in lines 176-183. For the verification of rKVAC85B, PCR was conducted using 1μl of plaque supernatant as the template. The initial denaturation was carried out at 95°C for 5 minutes, followed by 30 cycles of amplification at 95°C for 30 seconds, 57°C for 30 seconds, and 72°C for 3 minutes. The final extension was performed at 72°C for 5 minutes.

(ii) the authors should provide clear pictorial (cartoon) outlines of the immunogenicity and protective efficacy study protocols as part of the respective result figures, for greater clarity to the reader.

Reply: In response to your suggestion, we have added the mouse vaccination schedule to Figures 2A and 3A of our manuscript. This addition visually represents the timeline of the vaccinations administered to the mice, ensuring that the experimental progression and key phases are easily comprehensible to our readers.

(iii) the methodology for the bead-based cytokine analysis could be expanded to include the rationale for selecting these cytokines.

Reply: Thank you for your insightful comments. We agree that expanding the description of the rationale behind the selection of the 10 cytokines analyzed in our study is crucial for enhancing the transparency and understanding of our methodology. In response to your suggestion, we have updated lines 256-262 in the Materials and Methods section of our manuscript to include detailed explanations of why each cytokine was chosen and their biological significance. This addition will provide insights into the roles these cytokines play in immune responses and disease progression, and why they were considered critical markers for our research.

(iv) while MGIA is an interesting and powerful ex vivo assay to measure the efficacy of cellular immunity in controlling bacterial growth, inclusion of BCG as the target bacteria reduces its significance in this study that targets to evaluate the efficacy of new vaccine against two virulent strains of M. tuberculosis. This requires appropriate rationale or redoing it with virulent M. tuberculosis.

Reply: Thank you for your valuable comments. We have carefully considered your observations regarding the methodology of our study, particularly the suitability of using BCG as the target bacterium in the Mycobacterial Growth Inhibition Assay (MGIA). To address your concerns, we have referred to recent studies to enhance our response. According to the research conducted by Satria A. Prabowo et al. (2019) (PMID: 31114572), the RUTI vaccine, which is currently in clinical trial stages, significantly inhibits the growth of BCG and induces a shift in the monocyte phenotype in mice. Furthermore, related studies show that the results of growth inhibition assays using both BCG and Mtb as target bacteria for vaccines and therapeutics are consistent (PMID:27519524). This suggests that the outcomes of MGIA using BCG can have a meaningful correlation with those using Mtb. Additionally, research by O’Shea MK et al. (2018) (PMID: 36262883) analyzed growth inhibition after co-culturing blood from non-infected individuals, active TB patients, and individuals with latent TB infection with both BCG and H37Rv. The study found that the growth of the two strains was similarly influenced by the disease status. These findings support the validity of using BCG in MGIA as an effective method for evaluating the efficacy of tuberculosis vaccines. Based on these findings, we conclude that the use of BCG in our MGIA does not compromise the validity of our results compared to using virulent *M. tuberculosis* strains. We believe this substantiates the use of BCG in our experimental approach and provides reliable insights into vaccine efficacy.

3. Results:
The description of results and the respective figures need appropriate revision. For instance:
(i) the immunogenicity experiment seems to be conducted for all five groups together (Unvaccinated, BCG, rKVAC85B, rKVAC85B prime boost, and BCG- KVAC85B prime boost) as described in the M&M section (Line 150). However, results were depicted separately which makes it difficult to compare among all the groups. Animal numbers in each group for immunogenicity study was not mentioned, while for efficacy study it is mentioned as (n=5). Figure 2 C and D, and Figure 4 A and B bar diagrams should be provided with the error bars and appropriate statistics to show significance of the observed differences. The conc. of Ag85B protein that was used as the coating/stimulating agent not mentioned. Details of Ag85B epitope/peptides used for ELISPOT assay should be provided. Figure 5, IL-10 levels were depicted, typically this is a Th2 cytokine and known to enhance pathogen favoured response in TB, however, its heightened induction in the case of BCG prime rKVAC85B was not explained well. Information on number of samples, technical replicates should be provided.

Reply: Thank you for your invaluable feedback.

The primary objective of our study was to analyze immunogenicity across various vaccination strategies to identify the optimal vaccination regimen. Accordingly, we analyzed the prime vaccination group and the BCG post-boosting vaccination group in comparison with the PBS group and the BCG vaccination group. Additionally, for the multifunctional T cell analysis results presented in Figures 2C, 2D, 4A, and 4B, cells collected from all experimental groups were pooled for analysis. This pooling approach meant that it was not feasible to perform statistical analysis on individual groups. Consequently, we were unable to include error bars in these figures, and we kindly request your understanding in this matter.

And, we have revised the information regarding the materials used for coating and stimulation in our immunological assays to enhance clarity and detail in our manuscript. Specifically, for the ELISA assays, we utilized *E. coli*-expressed Ag85B protein sourced from abcam (MA, USA), applied at a concentration of 100 ng/well. In the ELISPOT assays, stimulation was performed using a set of 79 overlapping peptides of Ag85B (15-mers with 11 amino acid overlaps) obtained from JPT (Berlin, Germany), also at a concentration of 100 ng/well. These methodological details have been comprehensively updated and are now explicitly stated in the Materials and Methods section, specifically at line 255.

We understand your concerns about IL-10's role as a Th2 cytokine and its implications in tuberculosis. The increase in IL-10 following rKVAC85B administration is indicative of the vaccine’s ability to balance immune responses—moderating inflammation and preventing tissue damage while ensuring the immune system remains effective and controlled. This response is characteristic of the immune modulation often seen with vaccinia virus vectors, which can provoke both pro-inflammatory and anti-inflammatory cytokine production, including IL-10 (PMID:15919891, PMID: 29661092). Such a balanced immune response, where activation is sufficient to combat pathogens without causing excessive inflammation, is crucial for the long-term effectiveness and safety of the vaccine (PMID:30129209, PMID: 38251210).

We will provide a more detailed discussion on these aspects in the revised manuscript to clarify the role of IL-10 in tuning the immune response, ensuring both the potency and safety of rKVAC85B. We appreciate your insights, which are instrumental in enhancing the depth of our study.

(ii) as pointed out above, use of BCG as the target pathogen in the MGIA requires justification.

Reply: Thank you for your comments. The use of BCG as the target bacterium in our MGIA is based on several studies. Prabowo et al. (PMID: 31114572) found that the RUTI vaccine significantly inhibits the growth of BCG and alters monocyte phenotypes in mice. Research by O’Shea MK et al. (2018, PMID:36262883) also report consistent results in growth inhibition assays using both BCG and Mtb. These findings support the use of BCG in MGIA as a valid method for evaluating vaccine efficacy. We have included these details in the 'Discussion' section of our paper.

(iii) typically, in the mouse model of TB vaccine efficacy studies, both lungs and spleen CFU counts are provided. In this study, only lung CFU was shown. Spleen CFU data highlights how the vaccination would prevent dissemination of infecting pathogen to distant lymphoid organ. It would be better to show both lung and spleen CFU data. Further, in the absence of details of lungs homogenisation and plating protocol (which/how many lobes for what purpose, weight of lungs) it is difficult to appreciate the CFU data presentation as Log10 CFU/ml as weight/size of lungs varies between vaccinated and unvaccinated animals. Figure 7 needs major revision, it is appropriate to depict CFU data as per lungs or per gram of tissue, CFU data for all the groups in the study is warranted, labelling of the unvaccinated and infected group as normal is technically incorrect, should be revised.

Reply: Thank you for your feedback. We acknowledge the importance of presenting CFU counts from both the lungs and spleen in TB vaccine efficacy studies. To address the omission of spleen CFU data in our initial submission, we have added comparative data on the bacterial burden in the spleen post-challenge, as well as analysis of the degree of inflammation in lung tissue. These data are now included for the high-risk M. tuberculosis strain HN878 in Figure 7. Additionally, data concerning the bacterial burden and tissue pathology in the lungs for the H37Rv strain have been positioned in Figure S2. And, the CFU notation has been updated in the revised Figure.

4. Discussion:
The discussion could be strengthened by more critically comparing the results with existing studies on TB vaccines, particularly those using similar viral vectors, and explaining why the vaccine under study could be superior. Discuss the conundrum of heightened IL-10 response in the study. Also, highlight future research directions.

Reply: To strengthen the discussion section, we have more critically compared our results with existing studies on TB vaccines that use similar viral vectors, and have added explanations on why the vaccine under study could be superior. We have also addressed the issue of the heightened IL-10 response observed in our study and highlighted future research directions in the 'Discussion' section of our manuscript.

Minor Comments:

(i) Ensure consistency in the use of abbreviations throughout the manuscript.

Reply: We have thoroughly reviewed and ensured consistency in the use of abbreviations throughout the manuscript.

(ii) A few typographical and grammatical errors need correction for clarity and readability.

Reply: We have corrected the identified typographical and grammatical errors to enhance clarity and readability.

(iii) Ensure all references are up-to-date and formatted according to the journal's guidelines.

Reply: We have updated and formatted all references according to the journal's guidelines.
