# Supplementary material for: Efficacy and immunogenicity of rKVAC85B in a BCG prime-boost regimen against H37Rv and HN878 Mycobacterium tuberculosis strains
Source: PLoS One. 2025 May 14;20(5):e0322147. doi: 10.1371/journal.pone.0322147 (PMC12077692; doi:10.1371/journal.pone.0322147)
Supplement: Attachment — Submitted filename: Reviewers_comment_final_20250221.docx [file pone.0322147.s008.docx]

**Response to Reviewer`s Comments**

**Reviewer-1`s Comments to Author**

1. Although some parts have been improved, the revised version of the manuscript did not address the various criticisms raised by the reviewers, especially those that would have required novel experiments. In particular, the authors did not provide comparable data across all the different groups investigated (PBS, rKVAC85B, rKVAC85B boosting, BCG and BCG-rKVAC85B) that would have allowed a complete and correct comparison to assert that boosting with rKVAC85B enhanced the vaccine efficacy of BCG.

Reply: Thank you for your detailed comments and suggestions. We recognize the importance of providing comparative data across different groups to conclusively demonstrate that rKVAC85B boosting enhances the efficacy of the BCG vaccine, as you pointed out.

We conducted a comparative analysis of the specific IFN-γ secreting cell counts between the rKVAC85B prime-boost and the BCG-rKVAC85B boost groups. Although the experiments were conducted independently, they were performed using identical cell counts, enabling us to make direct comparisons based on these counts. Our analysis revealed that the BCG-rKVAC85B vaccination group showed significantly higher IFN-γ secreting cell counts in both the spleen and lungs compared to other groups, demonstrating the efficacy of the BCG-rKVAC85B boosting strategy.

Additionally, we conducted analyses of IgG ELISA OD values, finding that the mean OD value for the rKVAC85B group was approximately 0.875 (Fig 2B), whereas the BCG-rKVAC85B group exhibited a mean OD value of about 1.380 (Fig 3B). This indicates that the IgG responses in the BCG-rKVAC85B group were approximately 1.58 times higher than those observed in the rKVAC85B group. Moreover, the mean IFN-γ ELISPOT responses were about 437.33 spots per 10^6 cells for the rKVAC85B group and approximately 1688 spots per 10^6 cells for the BCG-rKVAC85B group, demonstrating that the BCG-rKVAC85B group exhibited IFN-γ responses approximately 3.86 times greater than those elicited by the rKVAC85B single vaccination (Fig S1). These findings underscore that the BCG-rKVAC85B vaccination not only significantly enhances IFN-γ responses but also boosts IgG antibody production, corroborating the effectiveness and necessity of BCG boosting in the immune modulation against tuberculosis.

Thank you once again for your valuable feedback. We look forward to your response and hope that our revisions meet your expectations.

**Fig S1.** Comparative IFN-γ ELISPOT Responses of Mice Immunized with rKVAC85B Alone and BCG Prime-rKVAC85B Boost

2. Also the lack of data on the basal levels of the various immunological parameters analyzed as well as the disparity of the vaccination protocols used do not allow to clarify what was actually modulated.

Reply: Thank you for highlighting the gaps in our dataset concerning the basal levels of immunological parameters and the variations in vaccination protocols. We acknowledge the importance of these baseline measurements, particularly in the poly-functional T cell response analysis, which unfortunately was not included in our initial study design.

The constraints of animal model experimentation, particularly under the stringent ethical guidelines that govern tuberculosis research, prevented us from conducting repeat experiments to address this oversight. Nonetheless, we have strived to provide a robust comparison by benchmarking against the existing BCG vaccine, which serves as the current standard in tuberculosis immunotherapy.

We recognize the critical nature of your feedback and will ensure more thorough baseline data integration in future experimental protocols to deepen the impact and clarity of our findings.

3. Furthermore, the histology figures of the lungs of the infected mice do not seem very consistent with what has been reported in panel D of Figure 7.

Reply: Thank you for your feedback regarding the histology figures in our manuscript, specifically the inconsistencies noted in panel D of Figure 7. We recognize that the limited presentation of histopathological data may have restricted our interpretative scope. Our findings indicate smaller lesion sizes predominantly in the BCG-rKVAC85B group, compared to larger lesions in the BCG and PBS groups. This suggests a variation in lesion severity aligned with the inflammation analysis in Figure 7D, though it is not directly conclusive. To provide clearer evidence, we included additional data in Figure 7C, showcasing more pronounced differences among the groups to support our conclusions.

**Fig 7. Comparative efficacy of BCG prime-rKVAC85B boost immunization against *M. tuberculosis* HN878.**
